# Biomechanical performance of a novel light-curable bone fixation technique

**DOI:** 10.1038/s41598-023-35706-3

**Published:** 2023-06-08

**Authors:** Peter Schwarzenberg, Thomas Colding-Rasmussen, Daniel J. Hutchinson, Dominic Mischler, Peter Horstmann, Michael Mørk Petersen, Stine Jacobsen, Tatjana Pastor, Michael Malkoch, Christian Wong, Peter Varga

**Affiliations:** 1 AO Research Institute Davos, Davos, Switzerland; 2 Department of Orthopedic Surgery, Hvidovre University Hospital, Copenhagen, Denmark; 3 Department of Fibre and Polymer Technology, KTH Royal Institute of Technology, Stockholm, Sweden; 4 Department of Orthopedic Surgery, Herlev and Gentofte Hospital, Hellerup, Denmark; 5 Department of Orthopedic Surgery, Rigshospitalet, Copenhagen University Hospital, Copenhagen, Denmark; 6 Department of Clinical Medicine, Faculty of Health and Medical Sciences, University of Copenhagen, Copenhagen, Denmark; 7 Department of Veterinary Clinical Sciences, University of Copenhagen, Copenhagen, Denmark; 8 Department for Plastic and Hand Surgery, Inselspital University Hospital Bern, University of Bern, Bern, Switzerland

**Keywords:** Biomedical engineering, Preclinical research, Fracture repair

## Abstract

Traumatic bone fractures are often debilitating injuries that may require surgical fixation to ensure sufficient healing. Currently, the most frequently used osteosynthesis materials are metal-based; however, in certain cases, such as complex comminuted osteoporotic fractures, they may not provide the best solution due to their rigid and non-customizable nature. In phalanx fractures in particular, metal plates have been shown to induce joint stiffness and soft tissue adhesions. A new osteosynthesis method using a light curable polymer composite has been developed. This method has demonstrated itself to be a versatile solution that can be shaped by surgeons in situ and has been shown to induce no soft tissue adhesions. In this study, the biomechanical performance of AdhFix was compared to conventional metal plates. The osteosyntheses were tested in seven different groups with varying loading modality (bending and torsion), osteotomy gap size, and fixation type and size in a sheep phalanx model. AdhFix demonstrated statistically higher stiffnesses in torsion (64.64 ± 9.27 and 114.08 ± 20.98 Nmm/° vs. 33.88 ± 3.10 Nmm/°) and in reduced fractures in bending (13.70 ± 2.75 Nm/mm vs. 8.69 ± 1.16 Nmm/°), while the metal plates were stiffer in unreduced fractures (7.44 ± 1.75 Nm/mm vs. 2.70 ± 0.72 Nmm/°). The metal plates withstood equivalent or significantly higher torques in torsion (534.28 ± 25.74 Nmm vs. 614.10 ± 118.44 and 414.82 ± 70.98 Nmm) and significantly higher bending moments (19.51 ± 2.24 and 22.72 ± 2.68 Nm vs. 5.38 ± 0.73 and 1.22 ± 0.30 Nm). This study illustrated that the AdhFix platform is a viable, customizable solution that is comparable to the mechanical properties of traditional metal plates within the range of physiological loading values reported in literature.

## Introduction

Traumatic bone fractures are often debilitating injuries that require surgical fixation for optimal healing. The frequency and economic burden of these injuries are expected to increase due to an increasingly elderly and more osteoporotic population^1^. Today, traditional metal implants are considered the clinical gold standard osteosynthesis material in surgical treatment for the majority of traumatic bone fractures^2^. Metal based implants have in many cases been shown to provide excellent biomechanical stability and healing potential^3,4^. However, in some clinical cases, metal-based materials are an inflexible solution lacking the versatility needed for various diverse fracture morphologies. Moreover, traditional metal plating has been shown to often induce side effects and complications such as stiffness, nonunion, hardware prominence, and tendon rupture^5^. This is especially true for tubular fractures in the hand and forearm, which are some of the most common skeletal injuries^4,6–8^, requiring early mobilization for sufficient bone healing^9^. While simple fractures in the hand may be treated nonoperatively with an external cast or splint, surgical treatment is often required for unstable or displaced fractures^4,10,11^.

A new osteosynthesis method, AdhFix, is under development to accommodate these clinical insufficiencies. AdhFix utilizes a light-curable polymer composite to provide highly customizable fixation solutions^12–15^. The method involves the insertion of metal screws into the bone fragments, followed by the in situ build-up of a polymer composite plate into a desired configuration. The biocompatible composite is formed from trifunctional allyl and thiol traizine-trione monomers and a high concentration of hydroxyapatite^13^. It is shaped in situ and rapidly cured into a rigid material through high energy visible (HEV) light induced thiol-ene coupling chemistry, giving surgeons a highly customizable fixation solution as an alternative to metal plating. In addition to its customizability, the composite used in AdhFix has been shown to have no soft tissue adhesions after 12 months in an in vivo rat model^13^.

To be a clinically viable solution, this novel system must be capable of withstanding physiologically relevant loading modes and magnitudes without failure. Initial investigations of the novel composite have been performed investigating its mechanical properties^13^, showing modulus values of 6.6 (0.2) GPa and maximum stress values of 69 (3) MPa. The composite was used with the AdhFix method to fixate fractures in ex vivo porcine and in vivo rodent bones, which revealed its suitability for stabilizing healing fractures and its lack of biosorbability over 12 months. However, large animal models such as sheep offer similar bone metabolism and skeletal size compared to humans, providing a representative surrogate to establish orthopedic methods and assess fixation stability^16^. In this study, we investigated the biomechanical performance of the AdhFix platform by comparing it to a traditional osteosynthesis solution, namely stainless steel locking metal plates and screws. Both platforms were compared by loading in four-point bending and torsion in an ex vivo ovine phalanx model of stable and unstable transverse fractures. Furthermore, since AdhFix is constructed in situ by hand, the reproducibility of each of the construct's morphology and the resulting biomechanics was investigated. The hypothesis of this study was that there would be no difference in the fixation stability between the AdhFix platform and traditional metal hardware in both bending and torsion in reduced and displaced fractures. A further hypothesis is that the width of the custom-made AdhFix patch would influence fixation stability in torsion, demonstrating the customizability of the platform.

## Materials and methods

### Sample preparation and group assignment

**Figure 1:**
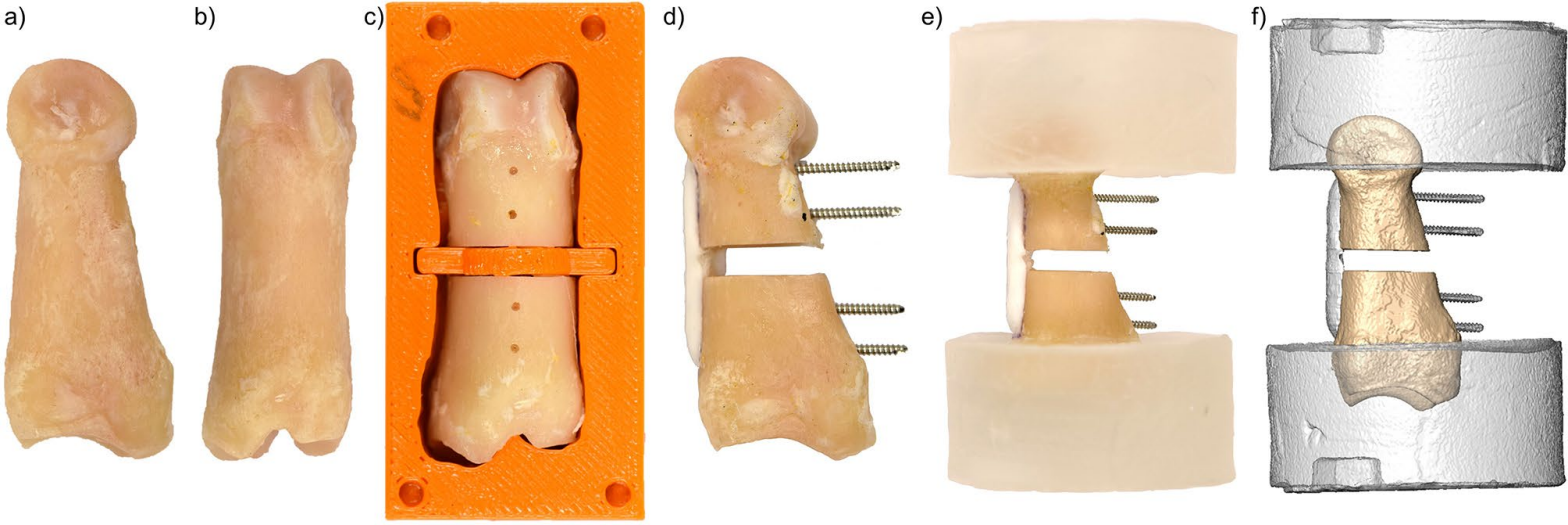
Workflow for the osteosynthesis of ovine phalanges. (**a**) Lateral view of an ovine phalanx. (**b**) Anterior view of an ovine phalanx. (**c**) Phalanx in 3D printed guide after drilling and cutting. (**d**) Osteosynthesized phalanx with AdhFix designated for torsion with a narrow patch (Group 5). (**e**) Osteosynthesized phalanx after PMMA embedding. (**f**) 3D rendering of osteosynthesized fracture model generated from micro-CT scan.

Forty-one ovine proximal phalanges were harvested after euthanasia from previously approved animal studies involving skeletally mature female sheep (age 3.59 ± 1.05 years; weight 73.28 ± 2.46 kg). No animals were sacrificed for the purpose of this study. The phalanges were excised, stripped of soft tissue, including the periosteum (Fig. 1a,b), and wrapped with gauzes soaked in ringer solution. The specimens were assigned to seven study groups (Table 1) according to osteotomy gap size (0 mm reduced fractures or 3 mm displaced fractures), fixation type (AdhFix or metal plate), loading modality (four-point bending or torsion) and fixation size (only for the Adhfix group in torsion; 6 mm width: narrow or 10 mm width: wide). The sample sizes were N = 3 in the metal-fixed groups and N = 8 in the Adhfix groups, as more variation was expected with this custom fixation. One sample was lost from Group 6 during subsequent steps, resulting in an N = 7.

**Table 1:** Testing group designation and details.

	Group	Fixation type	AdhFix patch size	Gap size	N =
	[−]	[AdhFix/metal]	[Narrow/wide]	[0 mm / 3 mm]	[−]
Four-point bending	1	AdhFix	Narrow (6 mm)	0	8
	2	AdhFix	Narrow (6 mm)	3	8
	3	Metal	–	0	7
	4	Metal	–	3	8
Torsion	5	AdhFix	Narrow (6 mm)	3	8
	6	AdhFix	Wide (10 mm)	3	7
	7	Metal	–	3	8

### CT scanning and 3D printed guides

After sample collection, all phalanges were scanned with high-resolution peripheral quantitative computed tomography (HR-pQCT) using an XtremeCT scanner (Scanco Medical AG, Brüttisellen, Switzerland) with an X-ray voltage of 60 kVp, X-ray current of 0.90 mA, and an isotropic resolution of 82 µm. The HR-pQCT scans were used to create specimen-specific 3D printed guides with cutting slots for osteotomizing the bones with an oscillating saw and drill guide support holes for drilling pilot holes for screws (Fig. 1c). The pilot holes were spaced 5 mm apart and 5 mm from the center of the osteotomy, with two on each bone segment to match the spacing on the metal plates that were used. To create these guides, each specimen's CT scan was imported into a 3D image processing software, Amira 3D (version 2021.1, Thermo Fisher Scientific), and a custom Python script was executed to create specimen specific cutting guides. The cutting guides were then 3D printed on a Stratasys F170 3D Printer (Stratasys Ltd., Rehovot, Israel) to create the physical guides to be used for experimentation. The volar side of the bone was aligned perpendicular to the cutting plane in bending while the dorsal surface was aligned for torsion. All AdhFix patches (groups 1, 2, 5, and 6) were 25 mm in length, independent of width.

### Pilot holes and osteotomies

The phalanges were placed in the cutting guides and four pilot holes were drilled through both cortices with a 1.1 mm drill bit (DePuy Synthes, Zuchwill, Switzerland). Next, either a single-cut transverse osteotomy or a 3 mm gap transverse osteotomy was performed depending on the group designation with an oscillating saw with a blade thickness of 0.6 mm. The final osteotomized bone is shown in Fig. 1c. Both drilling and cutting were performed under continuous irrigation with ringer solution to prevent dehydration, minimize the risk of bone damage, and to remove bone debris from the cutting regions.

### Osteosyntheses

**Figure 2:**
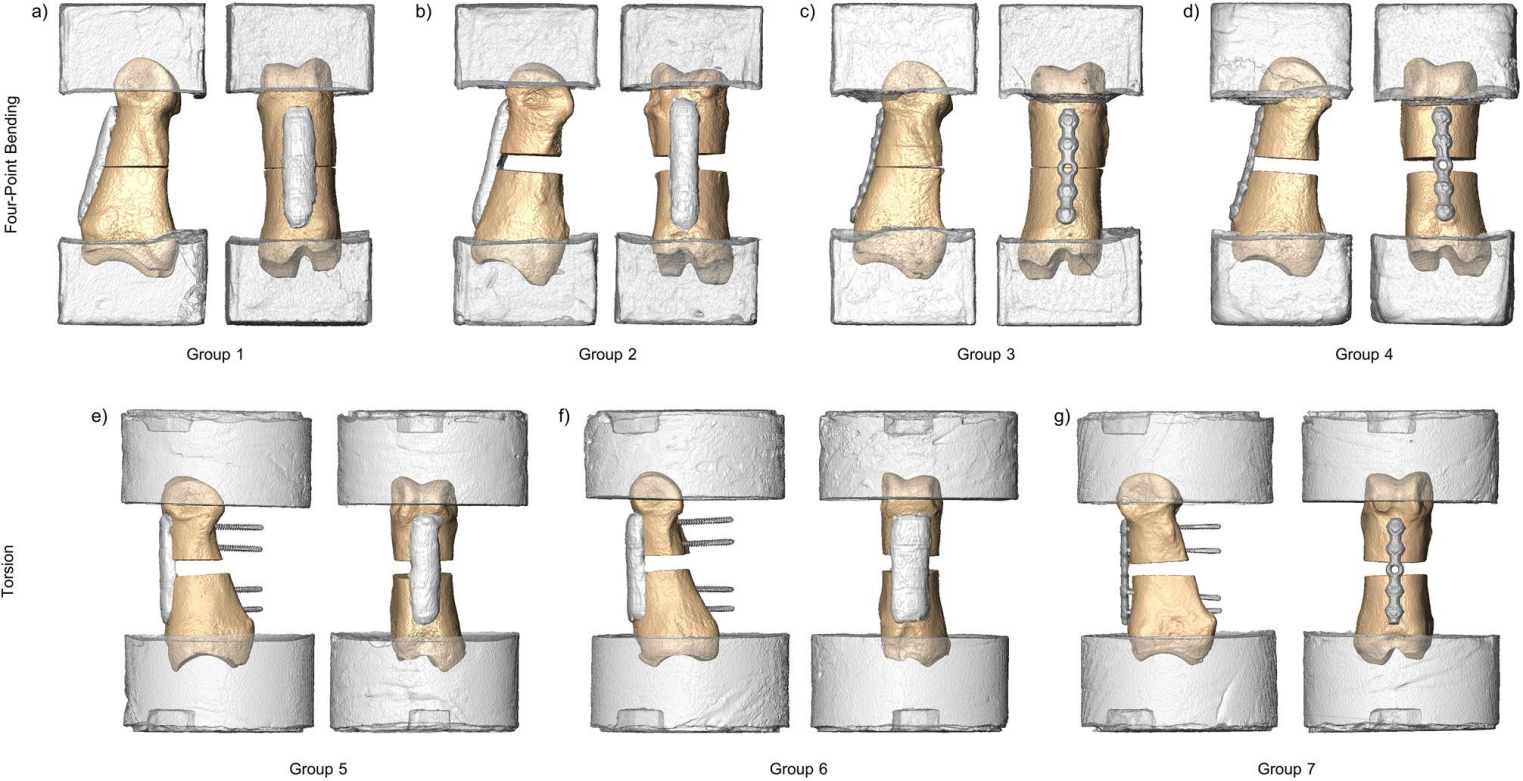
3D renderings of each testing group. (**a**) Group 1: AdhFix, four-point bending, 0 mm gap. (**b**) Group 2: AdhFix, four-point bending, 3 mm gap. (**c**) Group 3: AdhFix, four-point bending, 0 mm gap. (**d**) Group 4: Metal plate, four-point bending, 3 mm gap. (**e**) Group 5: Metal plate, torsion, 3 mm gap. (**f**) Group 6: AdhFix, torsion, 3 mm gap. (**g**) Group 7: Metal plate, torsion, 3 mm gap.

In the designated AdhFix groups, the light-curable customizable polymeric composite (Bonevolent™ AdhFix, Biomedical Bonding AB, Stockholm, Sweden) was applied using the method developed by Hutchinson et al.^13^ to create osteosyntheses mimicking bridge plating (Groups 1, 2, 5, and 6; Figs. 1d, 2). The AdhFix platform uses cortical screws to adhere the polymer composite to the bone, achieved by inserting stainless steel cortical screws (1.5 mm, DePuy Synthes) into the four pilot holes. In the torsion groups, the 25 mm screws were left uncut, however, in the bending groups, the pilot holes were measured with a depth gauge and the screws were cut such that they were flush with the volar surface of the bone. Next, the light-curable composite was applied around and under the screw heads using a syringe and spatula, and the screws were tightened against the bone surface. The composite was then cured with a high energy visible light source (Bluephase PowerCure LED lamp, Ivoclar Vivadent Clinical, Schaan Liechtenstein). The curing process included two 5-s pulses of 2000 mW/cm^2^ light from a 0.8 cm diameter light source. Composite was applied between the screws, connecting these attachment points to bridge the fracture and complete the initial composite layer. In the 0 mm gap models, the gap was reduced with slight pressure to ensure reduction. In the 3 mm gap model, a 3-D printed spacer was inserted into the guide to ensure proper spacing and provide a specimen specific contoured surface for the bridging composite to rest on and to avoid spillage into the gap (Fig. 1c). Once the initial bridging layer was established, a layer of polymer composite and a polyethylene terephthalate (PET) mesh (0.15 mm pores; 4 mm wide in narrow groups (Groups 1, 2, and 5) and 8 mm wide in the wide group (Group 6)) was added and cured into a second layer of the composite spanning the length of the patch. Finally, a third layer of the polymer was applied and cured on top of the mesh to completely encapsulate it. Examples of the AdhFix groups are illustrated in Fig. 2a,b,e,f.

Stainless steel locking plates (1.5 mm LCP Compact Hand, with 1.5 mm stainless steel locking screws, Depuy Synthes) were used for osteosyntheses on groups 3, 4, and 7 (Fig. 2c,d,g). Plates were cut to a 5-hole length from a 12-hole stock piece and the 1.5 mm locking screws were inserted through the plate holes and driven into the pilot holes until the screw heads locked to the plate.

In all groups, both in AdhFix and the metal plates, the surgical procedures and osteosyntheses were performed by the same orthopedic surgeon.

### Embedding

In the four-point bending groups (Groups 1–4), following osteosynthesis, the epiphyses of each bone were embedded in polymethyl methacrylate (PMMA) using a Teflon (PTFE) mold that resulted in 30 × 30 × 20 mm PMMA blocks (Fig. 2a–d).

Samples designated for torsion were embedded at the epiphyses in PMMA cylinders with a diameter of 60 mm with a 10 mm hex cavity aligned with the axis of the osteosynthesis to position and load the constructs (Figs. 1e–f, 2e–g).

### Patch thickness calculation

After embedding, all samples were scanned with the same CT scanner using identical settings as stated before. The AdhFix composite material is radio-opaque allowing for the average thickness of the patch to be calculated using a method of inscribed spheres from the CT scans (Fiji^17^, BoneJ plugin^18^).

### Mechanical testing

**Figure 3:**
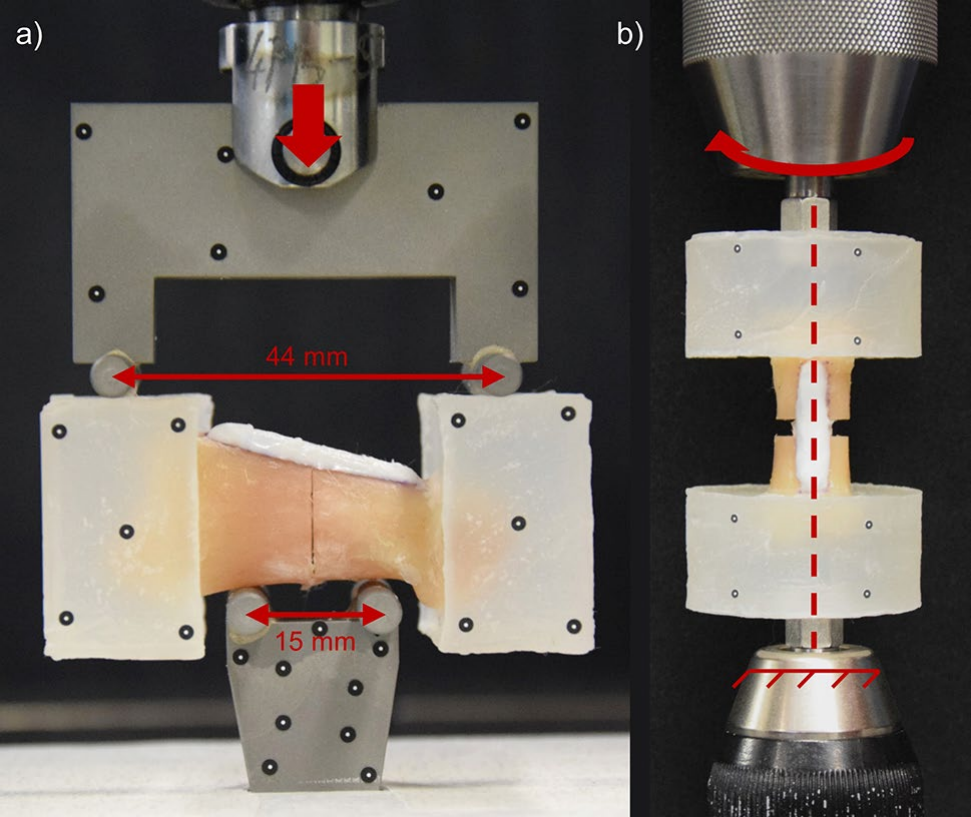
Mechanical testing setups. (**a**) Four-point bending testing setup. The upper contact rollers had a span of 44 mm while the lower contact rollers had a span of 15 mm. The fixture was loaded axially at a rate of 3 mm/min. (**b**) Torsion testing setup. The lower support was fixed while the upper support was rotated at a rate of 6°/sec. Both supports were 10 mm hexes aligned with the osteosynthesis by a hex cavity in the PMMA embedding.

For mechanical testing of the four-point bending constructs, the PMMA blocks were used as the bearing surfaces for the wide span (44 mm), while the volar surface of the phalanx was used as the bearing surface for the narrow span (15 mm; Fig. 3a). The four-point bending fixture was mounted to an electromechanical testing machine (Instron 5866, Norwood, MA, USA) with a 10 kN load cell. The samples were loaded in compression at a rate of 3 mm/min until failure of the osteosynthesis or a catastrophic bone failure in the phalanx. A stereographic camera system, the Aramis SRX (GOM GmbH, Braunschweig, Germany), was used to measure the displacement of the four-point bending fixture through the axis of rotation of the upper contact points. The bending moment was calculated from the applied force for the specific fixture that was used, and the maximum applied moment was queried for each specimen. In the AdhFix samples, bending stiffness was calculated as the slope of the linear region of the applied moment-displacement curve between 25 and 75% of the maximum applied moment in MATLAB 2020b (The MathWorks, Inc). In the metal-plated samples, the linear region was not in the range of 25–75% of the maximum load and was therefore selected manually. Furthermore, for the 3 mm gap metal-plated samples (Group 4), the deformation was large enough that the inner surface of the PMMA blocks impinged on the lower four-point bending fixture at higher loads. At this point, the assumed loading scenario was violated. Accordingly, the stiffness in these samples was evaluated in the region of free motion before impingement of the PMMA blocks to the lower bending fixture occurred according to markers on the PMMA blocks measured by the camera system.

In torsion, the hex cavity in the PMMA was utilized to align the axis of the osteosynthesis to an electromechanical testing machine (Instron 5943) and load the constructs (Fig. 3b). The constructs were loaded in torsion at a rate of 6°/second until construct failure or 30° of rotation was reached. Similar to four-point bending, the ARAMIS SRX system was used to measure the rotation of the two PMMA pots relative to each other using markers on the PMMA surface. Torque and angular displacement were measured, maximum torque was queried for each specimen, and torsional stiffness was calculated as the slope of the Torque-Displacement curve from 25 to 75% of the maximum torque in MATLAB. In the metal samples, stiffness was calculated as the slope of the initial linear portion of the curve before plastic deformation occurred.

### Statistics

Descriptive statistics and One-Way ANOVAs were performed in SPSS 27 (IBM Corp. Armonk, NY, USA). Statistical significance was determined at a level of *p* < 0.05. All groups were normally distributed according to a Shapiro–Wilk test except one (Group 4 bending stiffness; *p* = 0.045), which was still analyzed using the same method due to the robustness of the one-way ANOVA tests to deviations from normality^19^. Results are reported as means and standard deviations unless otherwise stated. When testing group differences with the One-Way ANOVA test, homogeneity of variance was determined using Levene's test for equality of variance. Due to the homogeneity of variance not being met in all samples, a Welch modified ANOVA was performed with a Games-Howell post hoc test to determine group differences.

## Results

### Four-point bending

**Figure 4:**
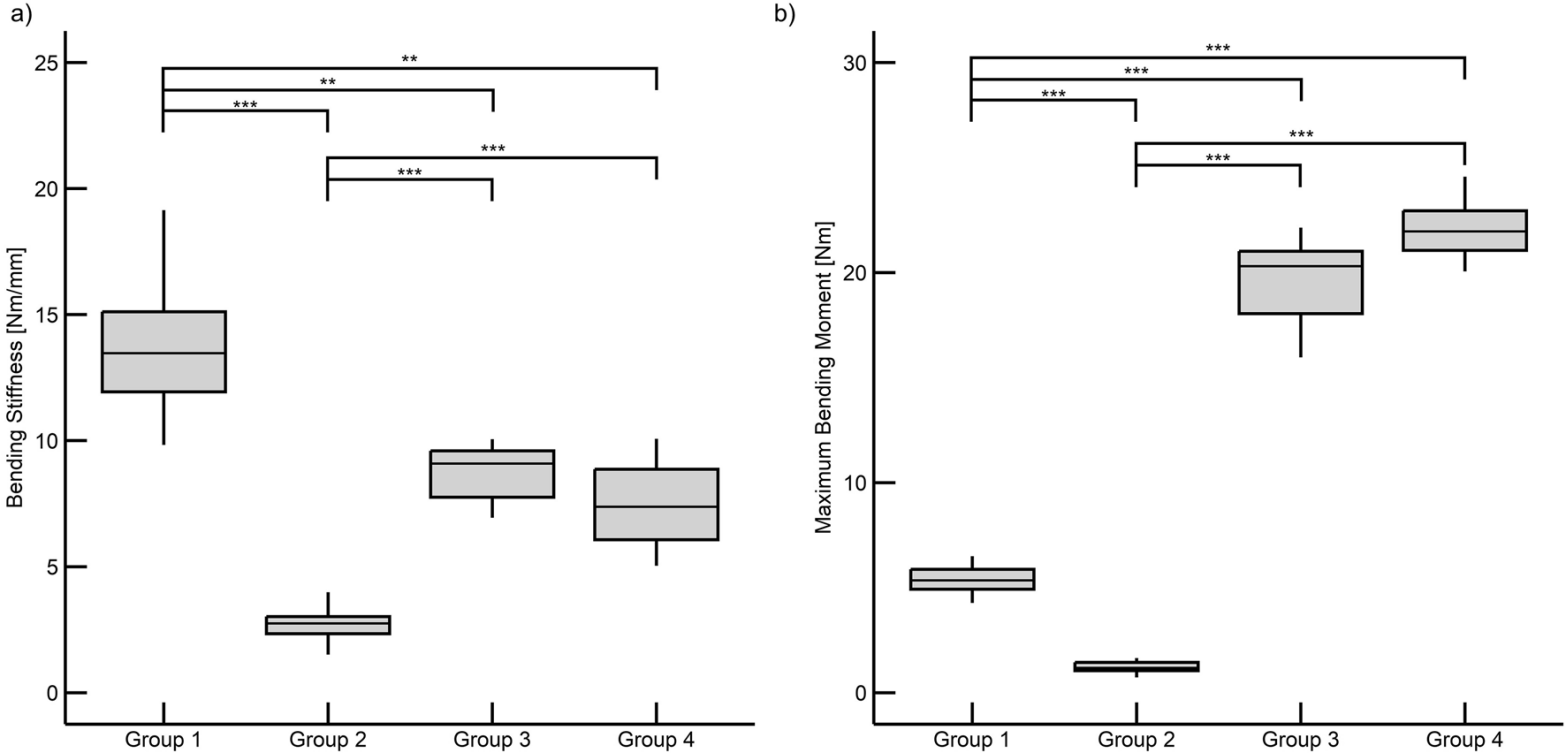
Box plots and scatter plots of the four-point bending results. Significance is denoted as *p* < 0.05 = *, *p* < 0.01 = **, and *p* < 0.001 = ***. (**a**) Bending stiffness results. (**b**) Maximum bending moment results.

The highest bending stiffness (13.70 ± 2.75 Nm/mm) was found in the 0 mm AdhFix group (Group 1), with the lowest (2.70 ± 0.72 Nm/mm) found in the 3 mm AdhFix group (Group 2). The 0 mm metal plate group (8.69 ± 1.16 Nm/mm; Group 3) and the 3 mm metal plate group (7.44 ± 0.1.75 Nm/mm; Group 4) fell in between. The modified ANOVA tests showed that all groups were significantly different from each other (*p* < 0.05, Fig. 4a, Table 2) except for the metal osteosyntheses groups (Groups 3 and 4) (*p* = 0.450).

**Table 2:** Four-point bending results and statistical significance values (*p*).

Group	Bending stiffness						Max bending moment					
	Mean	Std Dev	*p* values to each group				Mean	Std Dev	*p* values to each group			
[−]	[Nm/mm]		Group 1	Group 2	Group 3	Group 4	[Nm]		Group 1	Group 2	Group 3	Group 4
1	13.70	2.75	–	0.000	0.007	0.001	5.38	0.73	–	0.000	0.000	0.000
2	2.70	0.72	0.000	–	0.000	0.000	1.22	0.30	0.000	–	0.000	0.000
3	8.69	1.16	0.007	0.000	–	0.450	19.51	2.24	0.000	0.000	–	0.135
4	7.44	1.75	0.001	0.000	0.450	–	22.72	2.68	0.000	0.000	0.135	–

In the bending moment analysis, the highest maximum bending moment was observed in the 3 mm metal plate group (22.72 ± 2.72 Nm; Group 4), followed by the 0 mm metal plate group (19.51 ± 2.24 Nm; Group 3). The 0 mm AdhFix group (5.38 ± 0.73 Nm; Group 1) and the 3 mm AdhFix group (1.22 ± 0.30 Nm; Group 2) were lower. The modified ANOVA tests showed all groups were significantly different from each other (*p* < 0.05, Fig. 4b, Table 2) except for the metal osteosyntheses groups (Groups 3 and 4) (*p* = 0.135). Complete four-point bending results can be seen in Fig. 4 and Table 2.

### Torsion

**Figure 5:**
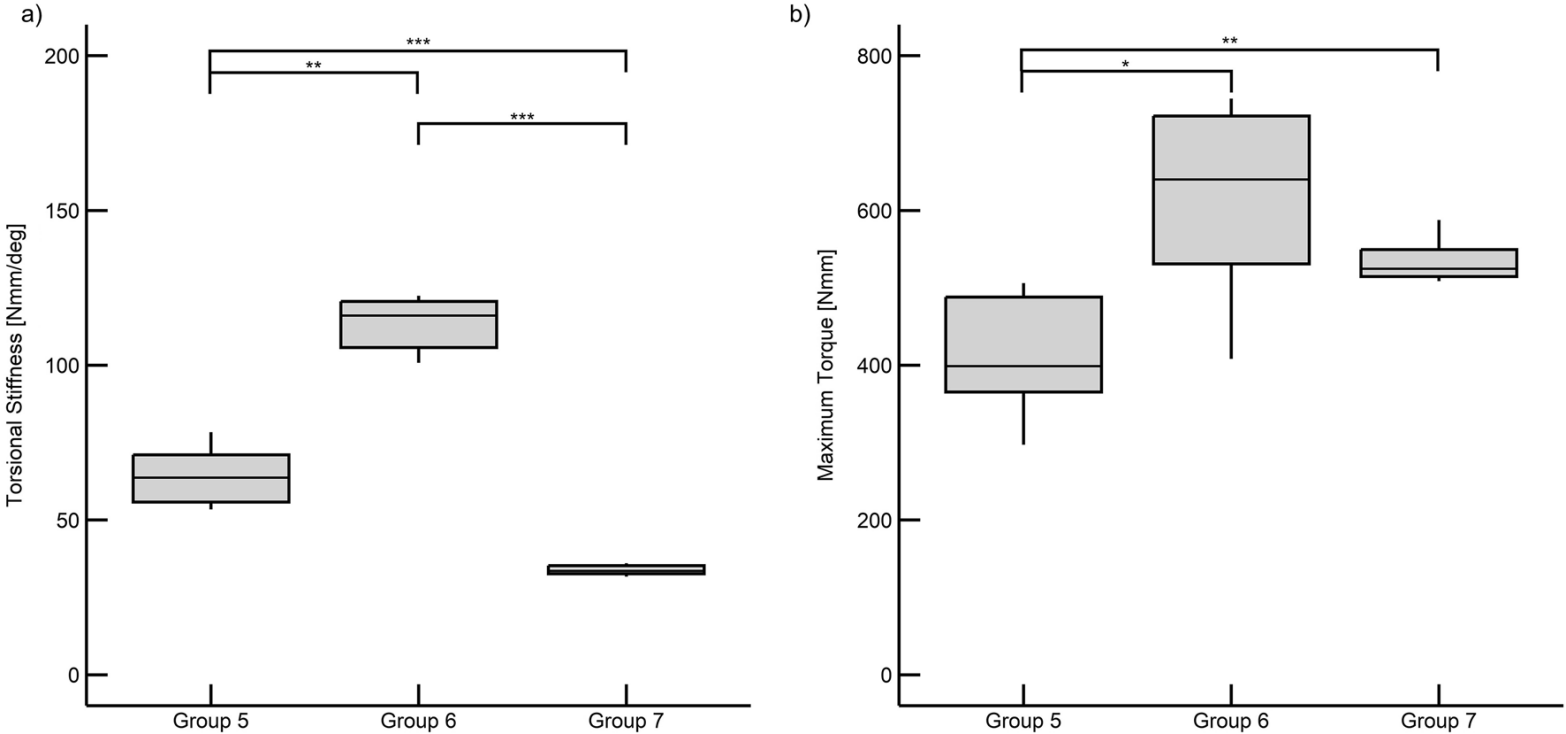
Box plots and scatter plots of the torsion results. Significance is denoted as *p* < 0.05 = *, *p* < 0.01 = **, and *p* < 0.001 = ***. (**a**) Torsional stiffness results. (**b**) Maximum torque results.

The highest torsional stiffness (114.08 ± 20.98 Nmm/°) was found in the wide AdhFix group (Group 6), with the lowest found in the metal osteosynthesis group (33.88 ± 3.10 Nmm/°; Group 7). The narrow AdhFix group (64.64 ± 9.27 Nmm/°; Group 5) fell in between. All groups were significantly different from each other (*p* < 0.05, Fig. 5a, Table 3).

**Table 3:** Torsional results and statistical significance values (*p*).

Group	Torsional stiffness					Max Torque				
	Mean	Std Dev	*p* values to each group			Mean	Std Dev	*p* values to each group		
[−]	[Nmm/°]		Group 5	Group 6	Group 7	[Nmm]		Group 5	Group 6	Group 7
5	64.64	9.27	–	0.002	0.000	414.82	70.98	–	0.013	0.006
6	114.08	20.98	0.002	–	0.000	614.10	118.44	0.013	–	0.304
7	33.88	3.10	0.000	0.000	–	534.28	25.74	0.006	0.304	–

Similarly, the highest maximum torque (614.10 ± 118.44 Nmm) was found in the wide AdhFix group (Group 6). However, the next highest torque (534.28 ± 25.74 Nmm) was found in the metal osteosynthesis group (Group 7), with the lowest (414.82 ± 70.98 Nmm) found in the narrow AdhFix group (Group 5). The modified ANOVA tests showed significant differences between groups. Group 5 was significantly different from the other two groups (*p* < 0.05, Fig. 5b, Table 3), while groups 6 and 7 were not significantly different from each other (*p* = 0.304).

### Patch thickness

The patch thickness of the four AdhFix groups (Groups 1, 2, 5, and 6) were 2.36 ± 0.32, 2.31 ± 0.30, 2.14 ± 0.26, and 2.13 ± 0.16 mm respectively. All groups met the assumption of homogeneity and the mean patch thicknesses were not significantly different between groups (*p* = 0.273). (Table 4).

**Table 4:** Mean patch thickness results.

Group	Mean patch thickness	
	Mean	Std Dev
[−]	[mm]	
1	2.36	0.32
2	2.31	0.30
5	2.14	0.26
6	2.13	0.16

## Discussion

This work demonstrated that the performance of the novel light-curable polymeric solution is biomechanically comparable to traditional metal fixators in certain situations. Our measurements help provide a better understanding of the potential of this novel construct. First, construct stiffness provided an understanding of the mechanics within the functional range of the osteosynthesis in both bending and torsion, where typical loading would occur. Second, the failure criterion of maximum bending moment and maximum torque provided an upper boundary as a design constraint to stay within. In a clinical setting, any failure or permanent damage would often require revision surgery or significant intervention to correct the failure. Finally, patch thickness provided a measurement of the uniformity of this customizable construct. By combining these three measures, with stiffness and failure investigated in two primary loading modes, a more complete understanding of the potential of AdhFix as an osteosynthesis material was gained.

The functional performance of AdhFix was demonstrated by the stiffness measurements. These measurements are representative of how the constructs perform in clinical use scenarios, not resulting in failure or permanent damage. The 0 mm gap AdhFix group (Group 1) demonstrated statistically superior bending stiffnesses to the other groups, including the metal-plated groups (Groups 3 and 4). In a clinical situation, this would mean a stiffer construct, which is required for a perfectly reduced fracture, which needs absolute stability for optimal healing^20^. However, the 3 mm gap osteotomy, which represents a comminuted fracture, fixed with AdhFix (Group 2), presented a less stiff construct than its metal counterpart (Group 4). As the construct was in contact with the bone, the effective working length was reduced to the gap width, as opposed to the distance between screws, creating even higher stresses in the AdhFix patch^21^.

**Figure 6:**
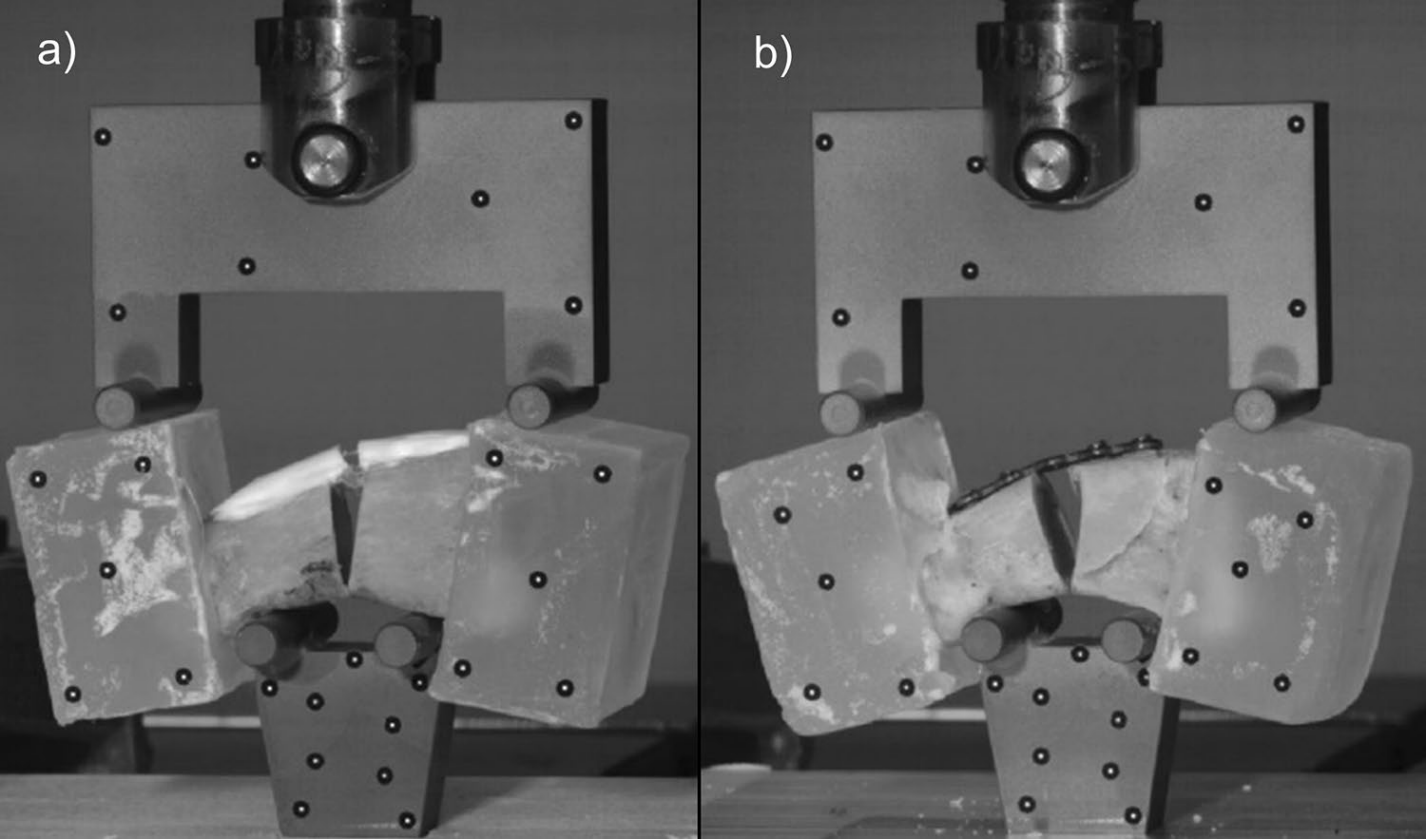
Photos of representative failure modes. (**a**) Failure of the AdhFix patch across the fracture gap. (**b**) Failure of the metal construct by catastrophic failure of the phalanx.

In contrast to bending stiffness, the metal constructs were consistently higher in maximum bending moment than the AdhFix patches in both the 0 and 3 mm gap osteotomy groups. However, the failure modes were not equivalent. The AdhFix constructs failed over the osteotomy at the fracture site and the metal constructs failed by a catastrophic failure of the bone at the screw insertion sites, which is not physiologically relevant (Fig. 6). This behavior could be attributed to two factors. First, the more brittle nature of AdhFix, compared to stainless steel, resulted in fracturing in the bridging construct in the AdhFix groups as opposed to around the screws in the metal groups, i.e. the metal plate and locking screw construct is stronger than the bone itself. This brittle behavior is also seen in the earlier failure of the AdhFix specimens with a gap when compared to their metal counterparts. This highlights the importance of adequate reduction and support in the fracture gap when using AdhFix. Second, the failure behavior in the metal constructs is an extreme case that far exceeds the expected biomechanical limits and requirements of the bone during normal rehabilitation exercises. Current literature reports external loads of up to 48 N applied to the hand during typical daily activities^22,23^. Furthermore, the average max bending moment in Group 2 (AdhFix: 3 mm gap in bending), which is the lowest performing AdhFix group, is equivalent to 168.5 N applied to the four-point bending fixture, or 84.2 N on each support. While these forces are not directly comparable due to the differences in loading modality, these values provide an estimate of the magnitude of physiological loads applied to a human phalanx. Therefore, the results of the AdhFix patches made in this study suggests that they would be able to sustain sufficient loads for rehab exercises and even daily biological use, even if they are statistically inferior to the metal constructs in maximum bending moment. Studies that determine the biomechanical load applied to a specific bone during normal rehabilitation exercises are warranted.

In torsion, the equivalent functional performance measure of the constructs is torsional stiffness. This measure showed that both the narrow and wide AdhFix groups (Groups 5 and 6) were statistically stiffer than the metal constructs (Group 7; *p* < 0.05) when tested in torsion with a 3 mm gap osteotomy. Furthermore, from an application standpoint, the narrow (Group 5) and wide (Group 6) groups were significantly different from each other, demonstrating that the constructs can be tuned for the specific mechanical requirements of a fracture scenario. This result is very important in a clinical setting as the rotation of the construct induces more shear strains which have been shown to inhibit bone growth^24^. In a clinical situation with complex fracture morphology, screw placement can be limited, and given the same screw placement, this in situ customizable patch can be widened to increase fracture stability using the same screw placement. Additionally, maintaining rotational alignment in phalanx fractures is an important surgical requirement to ensure functionality and prevent functional impairment^9,25^.

The final biomechanical measure of maximum torque demonstrated that while the narrow patch (Group 5) was statistically lower than the metal construct (Group 7; *p* < 0.05), the wide AdhFix patch (Group 6) and the metal plate were not significantly different (*p* = 0.304). This illustrates that in torsion AdhFix can be constructed to be equivalent to metal hardware in maximum torque.

The patch thickness measurements showed that the constructs were highly consistent, without any significant difference between groups. While this is not indicative of similar mechanical properties, it is important for the application process. In this study, the surgeon was assisted by a specimen-specific guide for both drillings of the screw holes as well as cutting the osteotomy. This aid reduced additional variations from the experiment to focus on the biomechanics of the AdhFix patch. Further studies of the surgical application are warranted to provide insight into the reproducibility of the customizable solution.

This study was not without limitations. The primary limitation in this study is the unknown loading scenario of the human phalanx, both during rehabilitation exercises as well as normal biological use. Ideally, it would be best to mimic these loading conditions, however, analyzing the constructs in both four-point bending as well as torsion captures much of the loading that is present in vivo in the phalanx. Additionally, the testing conducted in this study was monotonic until failure, whereas cyclic testing could be more relevant for clinical use. Future studies to better quantify biological loading and assess these loads cyclically would advance the understanding of the AdhFix platform as well as increase the relevance of this work. Furthermore, this study demonstrates intra-operative consistencies by a single surgeon, with inter-operative analysis of the AdhFix platform warranted in future studies.

In conclusion, AdhFix is a promising novel technology that provides a readily customizable solution for fracture fixation with the potential of reducing complications from soft-tissue adhesions. In this study, an ovine phalanx model was used to evaluate the biomechanical performance of the new fracture osteosynthesis technique compared to the current clinical gold standard of metal plating. The results presented in this study illustrate that the AdhFix platform has the potential to be a viable, customizable alternative to metal implants for fracture fixation, warranting further investigation in phalanges and similar bones.

## Data Availability

The raw mechanical testing data that the results are calculated from is available in the following public repository: https://doi.org/10.5281/zenodo.7985000.

## References

[1] Wu A-M (2021). Global, regional, and national burden of bone fractures in 204 countries and territories, 1990–2019: A systematic analysis from the Global Burden of Disease Study 2019. Lancet Healthy Longev..

[2] Onishi T (2015). Predictors of postoperative finger stiffness in unstable proximal phalangeal fractures. Plast. Reconstr. Surg. Glob. Open.

[3] von Kieseritzky J, Nordstrom J, Arner M (2017). Reoperations and postoperative complications after osteosynthesis of phalangeal fractures: A retrospective cohort study. J. Plast. Surg. Hand Surg..

[4] Carpenter S, Rohde RS (2013). Treatment of phalangeal fractures. Hand Clin..

[5] Page SM, Stern PJ (1998). Complications and range of motion following plate fixation of metacarpal and phalangeal fractures. J. Hand Surg. Am..

[6] Cotterell IH, Richard MJ (2015). Metacarpal and phalangeal fractures in athletes. Clin. Sports Med..

[7] Brei-Thoma P, Vogelin E, Franz T (2015). Plate fixation of extra-articular fractures of the proximal phalanx: Do new implants cause less problems?. Arch. Orthop. Trauma Surg..

[8] Guerrero EM (2021). Complications of low-profile plate fixation of phalanx fractures. Hand (N Y).

[9] Haughton D, Jordan D, Malahias M, Hindocha S, Khan W (2012). Principles of hand fracture management. Open Orthop. J..

[10] Logters TT, Lee HH, Gehrmann S, Windolf J, Kaufmann RA (2018). Proximal phalanx fracture management. Hand (N Y).

[11] Hardy MA (2004). Principles of metacarpal and phalangeal fracture management: A review of rehabilitation concepts. J. Orthop. Sports Phys. Ther..

[12] Granskog V (2018). High-performance thiol–ene composites unveil a new era of adhesives suited for bone repair. Adv. Funct. Mater..

[13] Hutchinson DJ (2021). Highly customizable bone fracture fixation through the marriage of composites and screws. Adv. Funct. Mater..

[14] Kieseritzky JV (2020). DendroPrime as an adhesion barrier on fracture fixation plates: An experimental study in rabbits. J. Hand Surg. Eur..

[15] Arseneault M (2018). The dawn of thiol–yne triazine triones thermosets as a new material platform suited for hard tissue repair. Adv. Mater..

[16] Martini L, Fini M, Giavaresi G, Giardino R (2001). Sheep model in orthopedic research: A literature review. Comp. Med..

[17] Schindelin J (2012). Fiji: An open-source platform for biological-image analysis. Nat. Methods.

[18] Doube M (2010). BoneJ: Free and extensible bone image analysis in ImageJ. Bone.

[19] Maxwell SE (2004). The persistence of underpowered studies in psychological research: Causes, consequences, and remedies. Psychol. Methods.

[20] Perren SM (2002). Evolution of the internal fixation of long bone fractures. The scientific basis of biological internal fixation: choosing a new balance between stability and biology. J. Bone Joint Surg. Br..

[21] MacLeod AR, Pankaj P (2018). Pre-operative planning for fracture fixation using locking plates: Device configuration and other considerations. Injury Int. J. Care Injured.

[22] Purves WK, Berme N (1980). Resultant finger joint loads in selected activities. J. Biomed. Eng..

[23] Fowler NK, Nicol AC (1999). Measurement of external three-dimensional interphalangeal loads applied during activities of daily living. Clin. Biomech. (Bristol, Avon).

[24] Epari DR, Kassi JP, Schell H, Duda GN (2007). Timely fracture-healing requires optimization of axial fixation stability. J. Bone Joint Surg. Am..

[25] Lee JK (2020). Outcomes following open reduction and internal fixation in proximal phalangeal fracture with rotational malalignment. J. Hand. Surg. Asian Pac..

